# Plant microRNAs regulate the defense response against pathogens

**DOI:** 10.3389/fmicb.2024.1434798

**Published:** 2024-08-30

**Authors:** Changxin Luo, Nawaz Haider Bashir, Zhumei Li, Chao Liu, Yumei Shi, Honglong Chu

**Affiliations:** Center for Yunnan Plateau Biological Resources Protection and Utilization, College of Biological Resource and Food Engineering, Qujing Normal University, Qujing, China

**Keywords:** microRNAs, pathogens, abiotic stress, plant defense, interaction

## Abstract

MicroRNAs (miRNAs) are a class of small non-coding RNAs, typically 20–25 nucleotides in length, that play a crucial role in regulating gene expression post-transcriptionally. They are involved in various biological processes such as plant growth, development, stress response, and hormone signaling pathways. Plants interact with microbes through multiple mechanisms, including mutually beneficial symbiotic relationships and complex defense strategies against pathogen invasions. These defense strategies encompass physical barriers, biochemical defenses, signal recognition and transduction, as well as systemic acquired resistance. MiRNAs play a central role in regulating the plant’s innate immune response, activating or suppressing the transcription of specific genes that are directly involved in the plant’s defense mechanisms against pathogens. Notably, miRNAs respond to pathogen attacks by modulating the balance of plant hormones such as salicylic acid, jasmonic acid, and ethylene, which are key in activating plant defense mechanisms. Moreover, miRNAs can cross boundaries into fungal and bacterial cells, performing cross-kingdom RNA silencing that enhances the plant’s disease resistance. Despite the complex and diverse roles of miRNAs in plant defense, further research into their function in plant-pathogen interactions is essential. This review summarizes the critical role of miRNAs in plant defense against pathogens, which is crucial for elucidating how miRNAs control plant defense mechanisms.

## 1 Introduction

Plants interact with diverse microbiota, including bacteria and fungi, which can have beneficial, detrimental, or neutral effects on the plants (Mendes et al., 2013). In symbiotic interactions between plants and microorganisms, both partners mutually benefit. Plants provide microbes with carbon fixed through photosynthesis, in the form of root exudates and litter, while microbes enhance the plants’ resistance to biotic and abiotic stresses (Bais et al., 2006; Bonfante and Genre, 2010; Sun et al., 2021; Kong and Liu, 2022). Pathogens, such as bacteria, viruses, nematodes, and fungi, can cause diseases by manipulating host cells with protein effectors that disrupt vital cellular functions and suppress both adaptive and innate immune responses of the host (Wang W. et al., 2018; Ahmed et al., 2022; Dai et al., 2023). To defend against such threats, plants have evolved various defense mechanisms, including structural and chemical defenses, hypersensitive responses, and systemic acquired resistance (Wang et al., 2019). Specifically, these defense mechanisms involve microRNAs (miRNAs), which trigger self-protective reactions that are crucial for plants lacking specialized cells responsible for immune functions (Xie et al., 2017; Islam et al., 2018).

Small RNAs (sRNAs) in plants play crucial roles in regulating development, stress tolerance, and antiviral defenses (Kamthan et al., 2015). These sRNAs can be divided into two types based on their biogenetic pathways and functions: miRNAs and small interfering RNAs (siRNAs) (Islam et al., 2017). MiRNAs are noncoding RNAs that bind to complementary sequences on messenger RNAs (mRNAs), enabling them to regulate gene expression and inhibit various signaling pathways in eukaryotic cells (Bartel, 2004). Recent research has highlighted the involvement of miRNAs in a wide array of biological processes, including mammalian reproduction, fertility, human stress-related illnesses, interactions with the gut microbiota, and plant growth and stress responses. They also play a role in regulating key agronomic traits (Li et al., 2017, 2020; Du et al., 2019; Salilew-Wondim et al., 2020; Šečić et al., 2021). As of the latest release of the miRBase blog,^1^ there are 48,885 mature miRNAs cataloged from 271 different species, including *Arabidopsis thaliana* (428 miRNAs), *Medicago truncatula* (756), *Brachypodium distachyon* (525), *Oryza sativa* (738), *Triticum aestivum* (125), *Zea mays* (325), and *Solanum lycopersicum* (147) (Kozomara et al., 2019; Šečić et al., 2021). However, given the complex and evolving nature of plant–pathogen interactions, it remains challenging to generalize the specific role of each miRNA in pathogen defense based on the results reported in studies. This review discusses the critical role of plant miRNAs in modulating the plant’s defense response to pathogens, including how they enhance disease resistance by regulating the balance of plant hormones and gene expression. In addition, the complex mechanism of plant-pathogen interaction is summarized, providing a valuable reference for research in the fields of biotechnology and molecular biology.

## 2 Plant defense mechanism against pathogen attack

**FIGURE 1:**
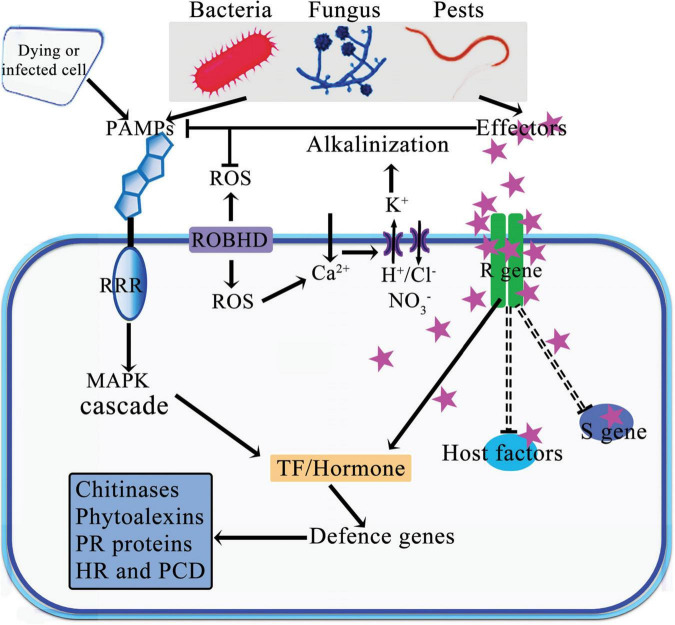
Schematic model of defense and attack strategies deployed during plant pathogen interactions. Plants activate their defense mechanisms in response to the detection of PAMPs and/or effectors from pests and pathogens by PRR proteins in plants. This recognition leads to the reprogramming of transcriptional regulation in defense gene and plant hormonal responses. Certain effectors bind to target genes or proteins, either inducing or reducing their expression or activity. Abbreviations: PAMPs, Pathogen-Associated Molecular Patterns; PRR, Pattern Recognition Receptors; R gene, Resistance gene; S gene, Susceptibility gene; RBOHD, Respiratory Burst Oxidase Homologue D; PR, Pathogenesis-Related; MAPK, Mitogen Activated Protein Kinases; HR, Hypersensitive Response; PCD, Programmed Cell Death; TF, Transcriptional Factor; ROS, Reactive Oxygen Species.

Viruses, bacteria, fungi, and nematodes are the primary agents of diseases in plants. These pathogens can directly destroy plant cells. For example, fungi destroy plant cells either by inserting the mycelium or hijacking host mechanisms to disrupt plant cell reproduction (Islam et al., 2017, 2018; Adnan et al., 2017). Over time, plants have evolved a complex array of defense mechanisms to adapt to environmental changes and resist pathogen invasion. These defenses are organized into multiple layers, including physical barriers, biochemical defenses, signal recognition and transduction, and the systemic acquisition of resistance (Figure 1; Jones and Dangl, 2006; Ngou et al., 2022). One of these sophisticated mechanisms involves the activation of self-defense responses through the involvement of miRNAs due to absence of some specialized plant cells with immune functions (Islam et al., 2018). In this mechanism, plant cells detect pathogen-associated molecular patterns (PAMPs) that trigger the primary immune response in the host plant, known as the PAMP-triggered immunity (PTI). To circumvent the PTI in plants, pathogens have also evolved specific effectors that disrupt the signal transmission associated with PTI (Thomma et al., 2011; Stam et al., 2014). In response, plants have developed a secondary defense system called effector-triggered immunity, mediated by various resistance (R) proteins. These R proteins specifically target and inhibit the spread of bacterial pathogen effectors, such as avirulence (avr) proteins, with high precision (Rouxel and Balesdent, 2010; Islam et al., 2018). Additionally, miRNAs play a role in modulating gene expression, either enhancing or suppressing it at both transcriptional and posttranscriptional levels. They respond to pathogen attacks by regulating gene expression through changes in hormonal signaling pathways including those of auxin, abscisic acid (ABA), and jasmonic acid (JA). Various miRNAs are actively involved in these defense mechanisms, playing crucial roles in plants’ response to different pathogens.

## 3 Plant miRNAs

### 3.1 Biosynthesis and functions of plant miRNAs and their role in gene expression regulation

**FIGURE 2:**
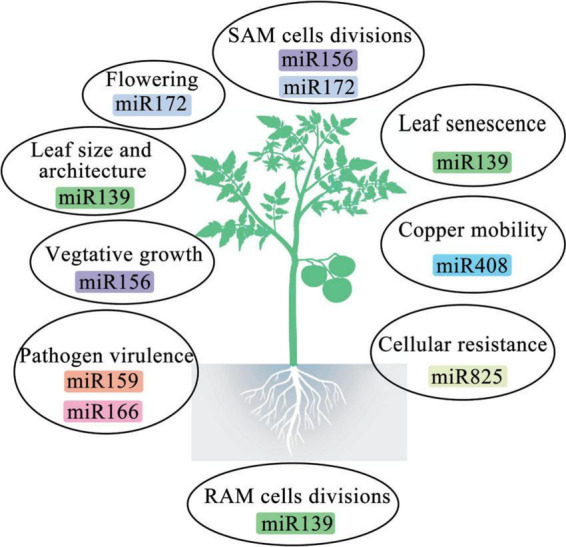
Roles of some miRNAs in plant life.

MiRNAs are a class of small RNA molecules that typically range in length from 20 to 24 nucleotides (nt), having crucial roles in the regulation of gene expression in eukaryotes (Friedman et al., 2009; Tarver et al., 2012). These miRNAs are expressed across various tissues and developmental stages, where they significantly affect disease resistance and stress tolerance. They are also implicated in macroevolutionary changes and are promising targets for bioengineering in domesticated species (Figure 2; Rubio-Somoza and Weigel, 2011; Tarver et al., 2013). In plants, miRNAs are produced from *MIR* genes primarily located in intergenic regions of genes that do not code for proteins. Conversely, the introns of protein-coding genes contain fewer *MIR* genes (Yang et al., 2012; Zhan and Meyers, 2023). These genes are transcribed by DNA-dependent RNA polymerase II (Pol II) into primary miRNAs (pri-miRNAs). Pri-miRNAs are single-stranded and polyadenylated RNA molecules. Their self-complementary nature allows them to fold into hairpin-like structures. These structures are then recognized and cleaved by the enzyme Dicer-like 1 to produce mature miRNA/miRNA duplexes (Reinhart et al., 2002; Zhan and Meyers, 2023). The mature miRNA duplex consists of the active miRNA strand, called the guide strand, and the complementary miRNA* strand, called the passenger strand (Liu et al., 2017). After methylation, both strands are transported to the cytoplasm where they integrate into the RNA-induced silencing complex (RISC). This complex is a ribonucleoprotein assembly comprising argonaute (AGO) proteins and small RNAs. Once integrated, the miRNA guides the RISC to either degrade the mRNA of the target gene or inhibit its translation, thus regulating gene expression (Voinnet, 2009; Zhan and Meyers, 2023). Typically, plant miRNAs are 21-nt long, although some are 20–22 nt long, with 23- or 24-nt miRNAs being less common (Chávez Montes et al., 2014; Axtell and Meyers, 2018).

### 3.2 The role of plant miRNAs in plant growth and development and their regulatory mechanisms

Plant miRNAs play essential roles in various aspects of plant growth and development, including organ morphogenesis, hormone function, signal transduction, and response to environmental stimuli (Figure 2; Zhao et al., 2019). There are two primary mechanisms through which miRNAs function in plants. The first mechanism is similar to that of siRNAs, where plant miRNAs are complementary to their target mRNAs; the 5′- untranslated region (UTR) of miRNAs interact with the open reading frame of the target mRNA. This interaction results in the cleavage and degradation of the target mRNA, preventing it from being translated. This mode of action is common in plants (Bartel, 2009; Khraiwesh et al., 2012; Hou and Zhao, 2013). The second mechanism involves miRNAs binding imperfectly to their target mRNAs and affecting translation, rather than transcription (Bushati and Cohen, 2007). In this process, miRNAs attach to the 3′-untranslated region (UTR) of the target mRNA. This binding either alters ribosome density on the mRNA or promotes degradation of the newly synthesized polypeptide chain, thereby inhibiting mRNA translation (Chen, 2004; Liu et al., 2022).

**FIGURE 3:**
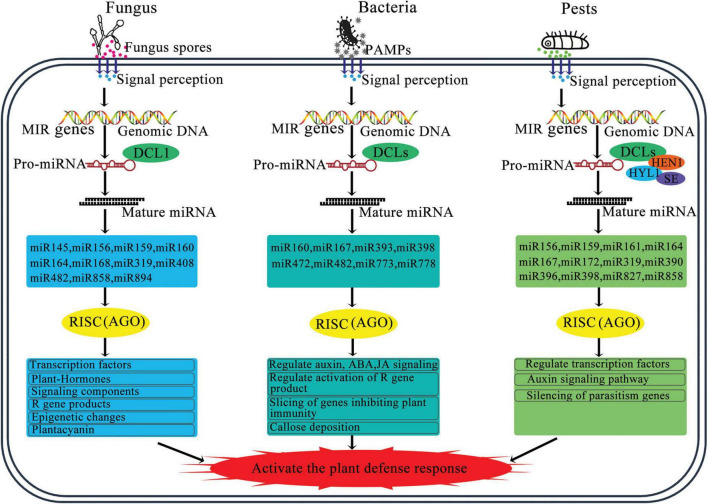
Schematic diagram of the regulatory role of miRNA in plant pathogen defense. The miRNA biogenesis and miRNA strand is incorporated into a member of the AGO protein family to form miRNA-induced silencing complex. By base-pairing, miRNA guides RISC to cleave target mRNA or repress target mRNA translation, and regulate other key processes. Abbreviations: PAMPs, pathogen associated molecular patterns; DCL, Dicer-like; AGO, argonaute; HYL1, hyponastic leaves 1; SE, serrate; HEN1, hua enhancer 1; RISC, RNAi-induced silencing complex.

A subset of miRNAs, known as intronic miRNAs, are present within the introns of genes they regulate, playing a significant role in modulating gene expression (Figure 3; Kim et al., 2015). The expression patterns of most intronic miRNAs align with those of their corresponding target genes. These intronic miRNAs can affect the expression of their target genes by affecting upstream transcriptional mechanisms. For example, they may downregulate transcriptional suppressors, thereby enhancing the expression of host genes (Lutter et al., 2010). In addition, some plant miRNAs can enhance the expression of target genes by binding to specific regulatory elements or proteins, which then adjust gene expression to cope with growth demands and environmental stresses (Jin et al., 2012; Zhao M. et al., 2015).

## 4 MiRNAs boost plant defense against phyto-pathogen

MiRNAs and effector proteins play a pivotal role in regulating and suppressing immunity in the interaction between plants and pathogenic microorganisms. In plants, miRNAs regulate the expression of immune receptor R genes through a mechanism known as RNA interference, helping to maintain a delicate balance between growth and defense. For instance, without pathogen infection, persistent high expression of the R protein could inadvertently trigger an autoimmune response, potentially inhibiting normal plant development. Consequently, a reverse regulatory mechanism involving miRNAs controls the expression of R genes to sustain background immune activity at an optimal level (Zhao and Guo, 2019; Cui et al., 2020). When soil-borne pathogens infect plants, miRNAs can be transported into fungal cells and play a trans-boundary disease resistance role. This suggests that miRNAs not only play a role within plant cells, but may also play a key role in the interaction between plants and pathogens (Zhao and Guo, 2019). Plant miRNAs not only function within plant cells, but can also be transported into bacterial cells, where they play a role in cross-kingdom RNA silencing. This trans-kingdom regulation represents an important signaling mechanism in the rapid response of plant-microbial interactions (Salvador-Guirao et al., 2018; Wang Z. et al., 2018).

Host-induced gene silencing (HIGS) is a technique based on RNA interference (RNAi) in plants in which sRNAs produced by the host plant are used to target the genetic material of intruders. This method has been successfully used to enhance plant resistance to various pathogens (Govindarajulu et al., 2015). In transgenic crops, artificial miRNAs (amiRNAs) have been effectively used to suppress gene expression in pests (such as aphids) and filamentous pathogens (such as oomycetes), amiRNA-mediated targeting represents a promising approach for increasing the effectiveness of HIGS (Zand and Innes, 2022). Specifically, the targeted use of amiRNAs against three soybean cyst nematode genes—J15, J20, and J23—resulted in reduced gene expression within nematode eggs in populations feeding on transgenic hairy roots (Tian et al., 2016). In another study, gene silencing in *Helicoverpa armigera* was achieved by introducing an amiRNA construct, derived from an insect miRNA precursor gene, into *Nicotiana benthamiana* plants. Despite the minimal processing of the altered amiRNA precursor by the plant’s Dicer-like enzymes, the ingestion of transgenic leaves by *H. armigera* effectively suppressed the target genes, leading to increased mortality and developmental irregularities in the insects (Bally et al., 2020). Insects, such as aphids, can absorb long double-stranded RNAs (dsRNAs) and hairpin RNAs, with miRNAs and siRNAs also capable of inducing gene silencing in these organisms. Notably, the gene silencing effects of amiRNAs derived from plant miRNA precursors and siRNAs generated from the expression of long dsRNAs can persist across multiple parthenogenetic generations in aphids (*Sitobion avenae*), continuing even after they return to feeding on nontransgenic plants (Pitino et al., 2011; Guo et al., 2014; Abdellatef et al., 2015; Zand and Innes, 2022). These findings highlight that HIGS, through the use of amiRNAs, not only boosts plant resistance to pests but may also produce lasting effects on pest reproduction and development by inducing gene silencing in both plants and insects.

## 5 MiRNAs act as regulators of gene expression in response to pathogen

The conserved canonical target genes of several dozen miRNA families are not directly involved in plant immunity. Instead, these genes regulate other critical processes, including development, hormone signaling, and nutritional homeostasis (Figure 3; Zhang et al., 2006; Jones-Rhoades, 2012; Asadi and Millar, 2024).

MiR156 of the miRNA family affects plant development and disease resistance by targeting SBP/SPL transcription factors (Poethig, 2013). Although manipulating the miR156-SPL pathway can enhance disease resistance in plants, it may have negative impacts on plant growth and yield. For example, in rice, reducing miR156 activity boosts resistance to a bacterial pathogen but at the cost of reduced yield. However, this trade-off can be mitigated by expressing an SPL homolog (ideal plant architecture1) under a pathogen-inducible promoter, which improves both resistance and yield (Liu et al., 2019).

MiR168 triggers cleavage of AGO1 mRNA by binding to the 3′ UTR of AGO1 mRNA, thereby reducing AGO1 protein synthesis (Vaucheret et al., 2006). In rice, miR168 silencing was reported to enhance yield, alter flowering time, and increase plant resistance to the rice blast fungus *Magnaporthe oryzae* (Wang et al., 2021). Genetic studies have indicated that the antiviral function of another AGO protein, AGO18, relies on its ability to sequester miR168, thereby reducing the repression of AGO1, which is crucial for antiviral RNAi (Wu et al., 2015).

MiR164 targets a family of genes encoding NAC transcription factors, which are involved in numerous biological processes (Sieber et al., 2007). In *Arabidopsis*, an miR164-regulated NAC has been suggested to promote programmed cell death, associated with the hypersensitive response, a plant’s localized defense mechanism (Lee et al., 2017). However, in rice, overexpression of miR164 suppressed immunity against the rice blast fungus *M. oryzae* (Wang Z. et al., 2018). The role of numerous miRNAs in plant defense response against pathogens has been documented (Table 1). The interaction between rice and *M. oryzae* has been most widely investigated. In addition to miR164 and miR168, other miRNAs including miR160, miR167, miR319, and miR398 have been reported to play a role in rice plants’ immune response to *M. oryzae* (Li et al., 2014; Zhang et al., 2018; Zhao et al., 2019). While miR167 and miR319 act as negative regulators of the immune response, miR160 and miR398 function as positive regulators, enhancing the plant’s ability to defend against pathogen invasion (Asadi and Millar, 2024).

**TABLE 1:** Characteristics of miRNAs against various pathogens.

miRNA	Target	Function	Related function against pathogenic bacteria	Reference(s)
miR156	SPL2, SPL3, and SPL10	Transcription factor	miR156/SPL9 regulates the accumulation of reactive oxygen species and the immune response, enhancing resistance of rice plants to brown planthopper through the silencing of miR156.	Mao et al., 2017 Ge et al., 2018 Yin et al., 2019
miR160	ARF10	Signaling transduction	Antagonizes the crosstalk between SA-mediated pathogen defense pathways and auxin-mediated growth processes.	Natarajan et al., 2018
miR164	NAC1	Auxin signaling and root development	NAC4 promotes pathogen-induced cell death under negative regulation by miR164.	Lee et al., 2017 Zeng et al., 2022
miR167	ARF8	Signaling transduction	Play crucial roles in regulating perception and signaling of auxin.	Ulmasov et al., 1999 Jeyaraj et al., 2019
miR168	AGO1	siRNA biogenesis	Targets ARGONAUTE1 and confers resistance against *Botryosphaeria dothidea* infection by altering defense responses.	Yu et al., 2017
miR390	ARF	siRNA biogenesis	Regulates *Nicotiana attenuata*’s response to *Manduca sexta* herbivory; modulates anthracnose resistance in apple.	Pradhan et al., 2021 Shi et al., 2022
miR319	TCP	GA signal transduction	The miR319/TCP4 module affects the expression of genes involved in the synthesis of JA and the endogenous JA concentration in leaves, thus playing a role in resistance to root-knot nematodes.	Zhao W. et al., 2015
miR393	TIR1/F-box AFB	Hormone response and others	Plays a role in the response of *Arabidopsis* to bacterial pathogens via recognition of pathogen-associated molecular patterns. It aids in pathogen resistance by downregulating the transcripts of auxin receptor genes.	Navarro et al., 2006; Wong et al., 2014
miR394	LCR	JA synthesis pathway	Negatively regulates the resistance of *Arabidopsis* to *B. cinerea* infection by targeting leaf curling responsiveness; it is also involved in the process of plant resistance to *Sclerotinia sclerotiorum* infection.	Tian et al., 2018 Zhang et al., 2021
miR398	CSD1, CSD2, CDS3, and CCS		Participates in an inverse regulatory mode against bacterial and fungal pathogens, and it may also be involved in the defense mechanism of plants against *Verticillium dahliae* infection.	Yang et al., 2021 Mei et al., 2022
miR472	NBS-LRR		Transgenic plants overexpressing miR472 exhibited higher susceptibility to Pst DC3000. However, knockdown of miR472 increased the resistance of plants to this pathogen, contributing to pathogen-associated molecular PTI in plants.	Navarro et al., 2006 Zhou et al., 2014 Jiang et al., 2020
miR528	Accumulation of reactive oxygen species	AO	Negatively regulates viral resistance in rice. Transcriptional regulation of miR528 by OsSPL9 orchestrates antiviral response in rice.	Wu et al., 2017 Yao et al., 2019
miR773	MIM773/MET2		Acts as a negative PTI regulator against bacterial pathogens.	Li et al., 2010

MiR319 plays a crucial role in biological stress responses. In tomatoes, miR319 targets the gene TEOSINTE BRANCHED/CYCLOIDEA/PCF 4 (*TCP4*), which is involved in the plant’s resistance to root-knot nematodes. This interaction affects the expression of genes related to JA synthesis and alters the levels of endogenous JA in leaves (Zhao W. et al., 2015). In rice, the expression of miR319 can be specifically induced by an infection with *Aspergillus oryzae*, leading to the suppression of its target gene, *OsTCP21*. When rice plants overexpressing miR319b (miR319b-OE) are infected with *A. oryzae*, this overexpression results in the inhibition of key enzymes involved in JA synthesis, such as lipoxygenases 2 and 5, which impact the plant’s defense mechanisms (Zhang et al., 2018).

Plants have developed various strategies to defend against microbial pathogens, with many miRNAs specifically targeting resistance-related nucleotide-binding site-leucine-rich repeat (*NBS-LRR*) genes, which are crucial for pathogen recognition and defense response (Park and Shin, 2015). MiRNAs regulate the *NBS-LRR* gene family by targeting their conserved domains. For example, a recent study indicated that miR482 limits the defense capabilities of potato plants by suppressing *NBS-LRR* genes in response to *Verticillium dahliae* invasion (Yang et al., 2015). Similarly, in cotton plants, reduced levels of miR482 correlate with increased NBS-LRR transcript abundance, leading to enhanced resistance against *V. dahliae* (Zhu et al., 2013). In poplar, miR472a plays a critical role in defense against the fungi *Colletotrichum gloeosporioides* and *Cytospora chrysosperma* by targeting NBS-LRR transcripts, the *Arabidopsis* miR472-RDR6 silencing pathway modulates PAMP- and effector-triggered immunity through the post-transcriptional control of disease resistance genes (Zhou et al., 2014; Su et al., 2018). In tomato plants, infection by *Pseudomonas syringae* suppresses miR482, which in turn triggers the activation of *R* genes, thereby boosting the plants’ defense against the pathogen (Shivaprasad et al., 2012). These results suggest the role of conserved target genes of the miRNA family in other key processes beyond plant immunity, such as development, hormone signaling, and nutritional homeostasis. miRNAs such as miR156, miR168, and miR164 influence plant development and disease resistance by targeting specific transcription factors, and these miRNAs play a role as positive or negative regulators of the plant’s immune response.

## 6 MiRNA regulates hormone homeostasis in response to pathogen infection

Various plant hormones, such as auxins, salicylic acid (SA), JA, and ethylene, regulate defense responses triggered by pathogen invasion. Although auxin promotes plant development and provides carbon and nitrogen, it can enhance the pathogenic potential of biotrophic pathogens by suppressing SA-mediated defense mechanisms. However, miRNAs play a crucial role in mediating these responses (Figure 3; Zhang et al., 2011; Kulshrestha et al., 2020; Šečić et al., 2021). MiR393 was the first miRNA identified to be regulated under biotic stress, where a modified rapid amplification of cDNA ends assay confirmed that it cleaves the auxin receptors transport inhibitor response 1 (TIR1) and auxin signaling F-Box (AFB) proteins (Navarro et al., 2006). The expression of miR393 is triggered by flg22, a molecular pattern derived from bacteria, leading to the RNA-mediated inhibition of TIR1/AFB proteins and regulation of auxin signaling (Navarro et al., 2006). This expression usually correlates positively with disease resistance, as evidenced by increased resistance through overexpression and enhanced susceptibility when miR393 is suppressed (Robert-Seilaniantz et al., 2011; Asadi and Millar, 2024). MiR393 also affects the production of tryptophan-derived secondary metabolites, such as indole-3-acetic acid, camalexin (CL), and indole glucosinolates (IG). Both CL and IG have antimicrobial properties; CL aids in defense against necrotrophic fungi by disrupting their cell membranes, whereas IG is effective against insects, biotrophic pathogens, and bacteria (Sugawara et al., 2009; Zhao et al., 2002; Robert-Seilaniantz et al., 2011). Similarly, miR160 and miR167, which target auxin response factors (ARFs), regulate the auxin signaling pathway (Ulmasov et al., 1999). MiR160 plays a pivotal role in local defense and systemic acquired resistance during interactions with *Phytophthora infestans* by regulating the crosstalk between auxin and SA pathways through its target StARF10, affecting the plant’s defense response (Natarajan et al., 2018). A study reported that miR160 and miR167 that target ARFs were both induced by all of the three *Pseudomonas syringae pv. tomato* (*Pst) strains* at 6 h post infection (hpi). Possibly, miR160, miR167, or miR390/TAS3-derived trans-acting siRNAs (tasiRNAs) can regulate over 30% of the 23 ARFs found in *Arabidopsis*, which function as positive or negative regulators of auxin signaling. Additionally, studies have reported that miR393, which targets the auxin receptors TIR1, AFB2, and AFB3, was differentially regulated by Pst DC3000 (EV), Pst DC3000 hrcC and Pst DC3000 (avrRpt2) (Guilfoyle and Hagen, 2007; Zhang et al., 2011). MiR319a/TCP plays a role in GA signal transduction and trichome formation in *Populus tomentosa*. Overexpression of miR319a leads to decreased levels of the targeted TCP transcription factors, significantly increasing leaf hair density in *P. tomentosa* and effectively reducing damage caused by insects (Fan et al., 2020).

In tomato plants, miR394 is involved in the negative regulation of the biological stress response. Overexpression of miR394 led to the inhibition of its target gene, leaf curling responsiveness (*LCR*), which, in turn, suppressed the expression of genes involved in JA synthesis. These results indicated that miR394 overexpression reduces the tomato plants’ resistance to *P. infestans* (Tian et al., 2018; Zhang et al., 2021). In *Arabidopsis* infected with *Botrytis cinerea*, an increase in miR394 level was observed, resulting in the decreased expression of *LCR* (Jin and Wu, 2015). In *Brassica napus*, miR394 is predicted to play a role in defense against *Sclerotinia sclerotiorum* infection by interacting with the JA signaling pathway (Joshi et al., 2016). Similarly, in garlic plants infected by *Fusarium oxysporum f. sp. cepae*, the expression of miR394 showed an increasing trend and disrupted the JA-dependent defense mechanism by directly affecting LCR (Chand et al., 2016).

When rice plants are infected by viruses, the expression of miR528 is suppressed, which leads to increased activity of its target gene, L-ascorbate oxidase (*AO*), reducing the accumulation of AO-mediated reactive oxygen species (ROS), a critical component of the plant’s defense response (Wu et al., 2017). Additionally, the miR528-AO defense pathway is regulated by SPL9, which activates miR528 gene expression by binding to specific motifs in its promoter region (Yao et al., 2019). In *Arabidopsis*, miR773, a non-conserved miRNA, acts as a suppressor of defense responses by targeting methyltransferase 2 (MET2), a DNA methyltransferase involved in epigenetic regulation (Salvador-Guirao et al., 2018). Inhibiting miR773 activity using target mimics (MIM773) resulted in increased MET2 expression, thereby enhancing the plant’s immunity against fungal pathogens. This enhanced immune response involves multiple signaling pathways, including those of ethylene, JA, and SA, and is characterized by increased callose deposition and ROS production upon pathogen invasion (Pieterse et al., 2012; Salvador-Guirao et al., 2018).

## 7 Perspectives

Pathogens are a persistent threat to global crop production. MiRNAs are indispensable not only to plant growth, development, nutrition, stress response, and hormone signaling pathways but also in plant defense against pathogens (Yang et al., 2021). These small noncoding RNAs, typically 20 to 25 nucleotides in length, are abundant in numerous plant species and play significant roles in posttranscriptional regulation of gene expression. This regulation impacts plant growth, development, stress responses, and hormone signaling pathways (Song et al., 2019). Plants interact with microorganisms in ways that can be either beneficial or harmful. To combat pathogens, plants have developed a range of defense strategies, including structural and chemical defenses, hypersensitivity, and systemically acquired resistance. MiRNAs are integral to these responses, particularly in regulating plant hormone homeostasis during pathogen attacks. Hormones such as auxin, SA, JA, and ethylene are key players in plant defense mechanisms activated by pathogens (Asadi and Millar, 2024). MiRNAs modulate the plant’s defense responses by regulating critical elements within the hormone signaling pathways. Given their complex and diverse roles in plant defenses, further investigations into the role of miRNAs in plant–pathogen interactions are vital. Understanding how miRNAs control plant defense mechanisms can lead to new strategies for breeding disease-resistant plants and enhancing crop yields.

## 8 Conclusion

This review delves into the critical role of miRNAs in the regulation of plant disease resistance genes. miRNAs, small non-coding RNAs approximately 20–25 nucleotides in length, are part of the post-transcriptional regulation of gene expression that affects a variety of biological processes, including plant growth, development, stress responses, and hormone signaling pathways. This review highlights the adaptive response of plants to pathogen invasions, where miRNAs are involved in activating or suppressing the transcription of target genes that regulate the host plant’s defense mechanisms. It discusses the mutually beneficial relationship in symbiotic interactions between plants and microbes, as well as the array of defense mechanisms that plants have evolved, such as structural barriers, chemical defenses, hypersensitive responses, and systemic acquired resistance (SAR). During pathogen invasion, plant cells recognize the presence of the invader through specialized receptors, triggering a complex signaling cascade. MiRNAs contribute to this process by precisely controlling the levels of key regulatory proteins, ensuring a coordinated and effective defense response. For instance, miRNAs have been shown to regulate the homeostasis of plant hormones, such as JA, SA, and ethylene, which are crucial players in the defense against pathogens. By modulating the expression of genes involved in hormone biosynthesis, perception, and signaling, miRNAs help plants to maintain the delicate balance of these defense-related phytohormones. Furthermore, miRNAs can target transcription factors and other regulatory proteins, further integrating the defense response at the transcriptional and post-transcriptional levels. This multilayered regulation allows plants to mount a robust and adaptable defense against a diverse range of pathogens. In summary, miRNAs are essential regulators of plant defense responses, with a particular focus on maintaining hormone homeostasis during pathogen attacks. Understanding the intricate role of these small RNA molecules in plant immunity can provide valuable insights for developing improved disease-resistant crop varieties and enhancing sustainable agriculture.

It is essential for future studies to thoroughly investigate the role of miRNAs in the evolutionary struggle between hosts and the pathogens that attack them. For example, the mechanisms by which fungal pathogens release and transport small RNA effectors to plant hosts, along with the corresponding secretion of sRNAs by the host to trigger cross-kingdom gene silencing, are not yet fully understood. Opportunities for the development of new strategies and technologies aimed at enhancing pathogen resistance in crops are also being presented by miRNAs and their targets. For instance, recent advancements in genome editing techniques are being leveraged to genetically engineer miRNAs and their targets, enabling the development of pathogen-resistant crops. This trend is anticipated to continue expanding in the future. In particular, the CRISPR/Cas9 system could be employed to induce mutations in miRNAs that negatively regulate plant resistance mechanisms. This approach would allow for the disruption of miRNA-mediated suppression of plant defense pathways, potentially enhancing the plant’s ability to withstand various biotic stresses. Undoubtedly, the identification of additional miRNA-target modules will make miRNAs a focus of resistance breeding in crops and trees in the future. Furthermore, the application of novel genome editing tools will also contribute to the focus on miRNAs in resistance breeding (Tang and Chu, 2017; Yang et al., 2021).

## Footnotes

1
https://mirbase.org/
